# Joint associations of leisure-time physical activity and sitting time with emotional wellbeing, physical functioning and work ability: an occupational study among young and early midlife Finnish municipal employees

**DOI:** 10.1186/s44167-023-00034-4

**Published:** 2023-12-01

**Authors:** Jouni Lahti, Jatta Salmela, Anne Kouvonen, Ossi Rahkonen, Tea Lallukka

**Affiliations:** 1 Department of Public Health, University of Helsinki, Helsinki, Finland; 2 Finnish Institute for Health and Welfare (THL), Helsinki, Finland; 3 Faculty of Social Sciences, University of Helsinki, Helsinki, Finland; 4 Centre for Public Health, Queen’s University Belfast, Belfast, Northern Ireland

**Keywords:** Leisure-time physical activity, Sedentary behaviour, Exercise, Employees, Functioning, Work, Emotional wellbeing, Work ability, Sitting time

## Abstract

**Background:**

Physical inactivity and sedentary behaviour are major public health problems. We examined the individual and joint associations of leisure-time physical activity (LTPA) and total sitting time with emotional wellbeing, physical functioning and work ability in young and early midlife employees.

**Methods:**

Cross-sectional questionnaire survey data were collected in 2017 among 19–39-year-old employees of the City of Helsinki (response rate 51.5%). LTPA (including commuting PA) was converted into a metabolic equivalent (MET) index. We classified the participants into four groups according to PA recommendations and participation in vigorous intensity activities. Total sitting time was classified into three groups using tertile cut-points (5.5 and 8.5 h/day). For joint analyses, we truncated LTPA into three groups and sitting time into two groups yielding a six-category variable. Emotional wellbeing and physical functioning were measured using the RAND-36 questionnaire and work ability with a scale ranging from 0 to 100. Linear regression analysis was used to estimate adjusted means and their 95% confidence intervals (CIs). The analytical sample included 4544 participants (80% females).

**Results:**

Adjusting for age and sex, high sitting time (> 8.5 h/day) was associated only with poorer emotional wellbeing. LTPA, especially vigorous activity, showed clear positive associations with emotional wellbeing, physical functioning and work ability. For emotional wellbeing, the low activity groups with low (70.51, 95% CI 69.1–71.9) and high (67.5, 65.5–69.5) sitting time and the moderate activity groups with low (72.5, 71.3–73.7) and high (70.4, 68.6–72.1) sitting time had statistically significantly lower mean scores than the corresponding vigorous activity groups with low (74.9, 74.2–75.7) and high (72.7, 71.6–73.9) sitting time. For physical functioning, the vigorous activity groups with low (96.3, 95.8–96.7) and high (96.2, 95.5–96.9) sitting time had significantly higher scores than the other groups. For work ability, the vigorous activity groups with low (82.0, 81.3–82.6) and high (80.8, 79.8–81.8) sitting time had significantly higher scores than the other groups. Adjusting for covariates only slightly attenuated these associations.

**Conclusions:**

Promoting PA among employees can contribute to better physical and mental health as well as better work ability.

## Background

In contemporary Western societies, the need for physical effort in daily lives is often minimal, and sedentary behaviour is increasingly common. Furthermore, most people do not engage in physical exercise regularly even if the negative effects of physical inactivity on various health outcomes are well established [1]. Sedentary behaviour is usually operationalised as sitting time and refers to behaviours of low energy expenditure, that is, equal or less than 1.5 metabolic equivalents (METs) [2]. In contrast, physical inactivity means a lack of moderate-to-vigorous physical activity, that is, less physical activity than recommended for health benefits [2, 3]. Excess sedentary behaviour and low physical activity are associated with various adverse health outcomes such as obesity, diabetes, cardiovascular diseases [4], and mortality [5]. Sedentary behaviour and physical activity partly describe opposite ends of the same phenomenon; however, physical inactivity and sedentary behaviour also independently affect health [6]. In their meta-analysis, Ekelund and colleagues showed that a high volume of physical activity is needed to counteract the harmful effects of high sedentary time in relation to mortality risk [5]. Furthermore, in joint analyses, high sedentary time has been associated with increased risk of metabolic syndrome, independent of physical activity level [7]. However, functional health outcomes such as work ability have received less attention in joint analyses of sedentary behaviour and physical activity.

Work ability is a multidimensional concept that is traditionally seen as a balance of health and functioning with the demands of work [8]. Examining work ability and related functional health outcomes, such as physical and mental aspects of functioning, is highly relevant since populations are rapidly ageing. Longer working careers and maintaining good work ability over the course of one’s working career is increasingly important for both individuals and the economy. Work ability is highly age-related as health problems and decline in functioning become more common with age. However, decreased health and work ability can already be seen among younger employees [9] and, in particular, problems in mental health are increasing among them [10]. Problems in health [11] and work ability [12] increase the risk of sickness absence and permanent work disability. Physical inactivity and sedentary behaviour may have important contributions to this relationship. Lack of leisure-time physical activity (LTPA) is connected to poorer physical health and functioning [13, 14] and mental health [14, 15], and increased sickness absence [16, 17]. High sitting time, in turn, has been associated with poorer mental health [18] and more sickness absence [19]. A municipal employee cohort offers good opportunities for examining work ability and related functional health measures since a variety of both manual and non-manual occupations with different levels of education and salary are included. The aim of this study was to examine the individual and joint associations of LTPA and total sitting time with emotional wellbeing, physical functioning and work ability among 19–39-year-old municipal employees.

## Methods

### Study population

The study is part of the Helsinki Health Study [20]. Cross-sectional survey data were collected in 2017 among all 19–39-year-old employees of the City of Helsinki, Finland whose employment had lasted for at least 4 months and who had at least a 50% employment contract at the time of the data collection (n = 11,459) [20]. The City of Helsinki is the largest employer in Finland with approximately 38,000 employees. Survey data were collected mainly online via secure server (n = 3407), and postal questionnaires were mailed to those without email addresses or who did not respond online (n = 1704) [20]. A non-response analysis showed that the data are broadly representative of the target population with respect to, for instance, sociodemographic and work-related factors and health [20]. We excluded participants with missing information in the study variables (n = 321), the outliers (n = 222) and those who were unemployed or on long-term sick leave or disability pension (n = 34); thus, the final analytical sample included 3635 female and 909 male employees (in total, n = 4544). The sex distribution matches that of municipal employees in the target population [20] and the municipal sector in Finland in general. The ethics committee of the Faculty of Medicine, University of Helsinki gave their approval for the study protocol. Additionally, the City of Helsinki provided permission for the Helsinki Health Study.

### Functional health outcomes

We used two RAND-36 subscales –– emotional wellbeing and physical functioning [21] –– as measures of mental and physical health and functioning. The emotional wellbeing subscale includes five items, for instance, being nervous, feeling calm and peaceful and being happy, with six response alternatives ranging from ‘all the time’ to ‘not at all’. The physical functioning subscale includes ten items, for instance, vigorous activities (e.g., running), moderate activities (e.g., brisk walking), lifting and carrying groceries, climbing stairs, and how the health of the respondent limits these activities. Each item had three response alternatives: ‘yes, limited a lot’, ‘yes, limited a little’ and ‘no, not limited at all’. For both physical functioning and emotional wellbeing, standardised scores were calculated and transformed to a score between 0 and 100, with higher scores indicating better health [22]. Work ability was enquired with a single-item question asking respondents to estimate their current work ability on a scale ranging from zero to ten, zero indicating fully disabled to work and ten indicating work ability at its best. The single-item work ability score has shown comparability with the multi-item work ability scale [8]. The work ability score was scaled to range from 0 to 100 (as the other functional health outcomes).

### Leisure-time physical activity and sitting time

Weekly leisure-time and commuting physical activity were measured with a series of questions on four different intensities ranging from low intensity activity, such as walking, to vigorous intensity activity, such as running [13]. Approximate metabolic equivalent (MET) hours per week for the volume of leisure-time and commuting physical activity were calculated [2, 23]. We classified the participants into four groups according to volume of LTPA and according to their participation in vigorous intensity activities following previous procedures [13]: (1) high vigorous activity (over 80 MET-hours per week including vigorous activity), (2) vigorous activity (20–80 MET-hours per week or more including vigorous activity), (3) moderate activity (moderate intensity activity 20 MET-hours or more per week) and (4) low activity (under 20 MET-hours per week, equivalent to 5 h of walking). Sitting time was measured with a series of questions on sitting time in different domains separately (at home in front of screen or TV, at home reading, in vehicle, at work, elsewhere) during typical weekdays as recommended for evaluating total sitting time as accurately as possible in surveys [24]. Total sitting time was classified into three groups using tertile cut-points: (1) low sitting time (under 5.5 h per day), (2) intermediate (5.5–8.5 h per day) and (3) high sitting time (over 8.5 h per day). For joint analyses, we truncated LTPA into three groups, combining vigorous and high vigorous activity groups, and sitting time into two groups, combining low and intermediate groups, yielding a six-category variable.

### Covariates

The selection of covariates was based on our previous studies on similar topics [11, 13, 14, 16]. We included sex (male/female) and age (years). Marital status was dichotomised into cohabiting/married and others (unmarried, widowed and divorced). Having children was dichotomised into those having children and no children under 18 years of age in the household. Education was categorised into three groups: (1) upper secondary or vocational school or lower, (2) bachelor’s degree and (3) master’s degree or higher. Working status was dichotomised into those in full- or part-time work and those on family leave or studying. Current smoking was dichotomised into smokers (daily and occasional) and non-smokers (never smokers and quitters). Binge drinking was dichotomised into those using more than six portions of alcohol at least once a month and occasional binge drinkers (including non-drinkers). Body mass index (BMI) was included as a continuous variable and was computed based on self-reported weight and height (kg/m^2^). For mental strenuousness of work, a single-item question with four response alternatives (‘very light’, ‘rather light’, ‘rather strenuous’ and ‘very strenuous’) was included, asking how mentally strenuous the respondent considered their work. Mental strenuousness of work was dichotomised into mentally non-strenuous (‘very light’, ‘rather light’ and ‘rather strenuous’) and mentally strenuous (‘very strenuous’). For physical strenuousness of work, a single-item question similar to that for mental strenuousness was included and dichotomised into physically non-strenuous (‘very light’ and ‘rather light’) and physically strenuous (‘rather strenuous’ and ‘very strenuous’).

### Statistical methods

First, descriptive statistics (n, %) were calculated. Then, a general linear model was used to calculate adjusted means and their 95% confidence intervals (CIs) for emotional wellbeing, physical functioning and work ability. Means scores and their 95% CIs provide estimates of the levels of physical functioning and work ability as well as the statistical significance of the differences between the groups. A clinically significant difference for physical functioning and emotional wellbeing has been suggested at three points [25], but for the work ability score such a difference has not been evaluated. The individual analyses for sitting time and LTPA were adjusted for age and sex. In the joint analyses, Model 1 was adjusted for age and sex and Model 2 further for marital status, having children, education, smoking, binge drinking, BMI, and mental and physical strenuousness of work. SPSS 27 statistical package was used.

## Results

Table 1 shows the distribution of LTPA, sitting time and covariates separately for female and male participants. The mean age was 32 years, two-thirds of participants were married or cohabiting, and four out of ten had children living in their household. 33% of the participants had upper secondary or vocational education or less, 37% had completed a bachelor’s degree and 30% had a master’s degree or higher. The mean BMI was 25.2, 24% were current smokers and a similar proportion were binge drinkers. A third of the participants reported physically strenuous work and less than one-fifth reported mentally very strenuous work.

Of the participants, 17% were classified in the low activity group, 23% in the moderate activity group—females slightly more often than males—and 39% in the vigorous activity group. A fifth of the participants were in the high vigorous group; this was slightly more common among males. High sitting time was more common and low sitting time less common among males.

**Table 1:** The distribution of leisure-time physical activity, sitting time and covariates among participants

	Female	Male	All
	% (n)	% (n)	% (n)
**Total**	80.0 (3635)	20.0 (909)	n = 4544
**Leisure-time physical activity per week**			
High vigorous activity^1^	18.7 (681)	27.7 (252)	20.5 (933)
Vigorous activity^2^	38.3 (1394)	40.2 (365)	38.7 (1759)
Moderate activity^3^	25.2 (917)	15.7 (143)	23.3 (1060)
Low activity^4^	17.7 (643)	16.4 (149)	17.7 (792)
**Sitting time per weekday**			
Low < 5.5 h	39.3 (1430)	28.0 (254)	37.1 (1684)
Intermediate	32.1 (1166)	32.1 (292)	32.1 (1458)
High > 8.5 h	28.6 (1039)	39.9 (363)	30.9 (1402)
**Joint sitting physical activity groups**			
Low sitting^5^ & Vigorous activity^6^	41.2 (1499)	43.8 (398)	41.7 (1897)
Low sitting & Moderate activity	17.8 (647)	7.8 (71)	15.8 (718)
Low sitting & Low activity	12.4 (450)	8.5 (77)	11.6 (527)
High sitting & Vigorous activity	15.8 (576)	24.1 (219)	17.5 (795)
High sitting & Moderate activity	7.4 (270)	7.9 (72)	7.5 (342)
High sitting & Inactive	5.3 (193)	7.9 (72)	5.8 (265)
**Covariates**			
**Age in years** (mean)	31.9	32.6	32.0
**Highest education**			
Upper secondary or vocational school, or lower	31.5 (1145)	41.1 (374)	33.4 (1519)
Bachelor’s degree	38.6 (1403)	29.5 (268)	36.8 (1671)
Master’s degree or higher	29.9 (1087)	29.5 (267)	29.8 (1354)
**Marital status**			
Married or cohabiting	62.6 (2276)	63.0 (573)	62.7 (2849)
Not married or cohabiting	37.4 (1359)	37.0 (336)	37.3 (1695)
**Children**			
Yes	42.0 (1526)	38.2 (347)	41.2 (1873)
No	58.0 (2109)	61.8 (562)	58.8 (2671)
**Physical strenuousness of work**			
Strenuous	35.3 (1283)	24.8 (225)	33.2 (1508)
Non-strenuous	64.7 (2352)	75.2 (752)	76.8 (3036)
**Mental strenuousness of work**			
Strenuous	17.8 (648)	16.7 (152)	17.6 (800)
Non-strenuous	82.2 (2987)	83.3 (757)	82.4 (3744)
**Body mass index** (mean)	25.0	26.0	25.2
**Smoking**			
Smokers	23.1 (840)	24.6 (224)	23.4 (1064)
Non-smokers	76.9 (2795)	75.4 (685)	76.6 (3480)
**Binge drinking**			
At least once a month	18.3 (664)	42.4 (385)	23.1 (1049)
Occasional /non-drinkers	81.7 (2971)	57.6 (524)	76.9 (3495)
**Employment status**			
Full- or part-time work	88.9 (3233)	97.9 (890)	90.7 (4123)
Family leave or studying	11.1 (402)	2.1 (19)	9.3 (421)

^1^ > 80 MET-hours including vigorous activity

^2^ > 20–80 MET-hours including vigorous activity

^3^ 20–80 MET-hours moderate intensity activity

^4^ < 20 MET-hours

^5^ ≤ 8.5 h of sitting per weekday

^6^ > 80 MET-hours including vigorous activity

MET = approximate metabolic equivalent

The joint 6-category variable showed that over 40% of both female and male participants reported low (less than 8.5 h) sitting time per day and had at least 20 MET-hours per week of physical activity including vigorous activity. Of the participants, 18% were in the active vigorous group and had high sitting time, 16% were in the moderate activity group and had a low sitting time, and 12% were in the low activity group and had low sitting time. Only 6% were in the low activity group and had high sitting time, and 8% were in the moderate activity group and had high sitting time.

The individual association analyses showed that sitting time was not associated with physical functioning or work ability. High sitting time (> 8.5 h/day) was associated with poorer emotional wellbeing mean scores (71.1, 95% CI 70.3–72.0) than low (74.1, 95% CI 73.3–74.9) and intermediate (73.1, 95% CI 72.2–74.0) sitting times (Table 2). LTPA showed clear positive associations with all outcomes, that is, emotional wellbeing, physical functioning and work ability. In particular, vigorous activity and high vigorous activity groups had better functional health outcomes (Table 2).

**Table 2:** Emotional wellbeing, physical functioning and work ability mean scores and their 95% CI by leisure-time physical activity groups and sitting time adjusted for age and sex

	Emotional wellbeing	Physical functioning	Work ability
	Mean, 95% CI	Mean, 95% CI	Mean, 95% CI
**Leisure-time physical activity per week**			
High vigorous activity^1^	75.1, 74.0-76.1	95.7, 95.0-96.3	82.5, 81.6–83.4
Vigorous activity^2^	73.9, 73.1–74.6	96.5, 96.0-96.9	81.2, 80.5–81.8
Moderate activity^3^	71.8, 70.8–72.8	93.8, 93.2–94.4	78.8, 77.9–79.6
Low activity^4^	69.5, 68.3–70.7	92.6, 71.9–93.3	77.4, 76.4–78.5
**Sitting time per weekday**			
Low < 5.5 h	74.1, 73.3–74.9	95.1, 94.6–95.5	80.6, 79.9–81.3
Intermediate	73.1, 72.2–73.9	95.0, 94.5–95.5	80.6, 79.9–81.3
High > 8.5 h	71.1, 70.3–72.0	94.9, 94.4–95.4	79.3, 78.6–80.1

^1^ > 80 MET-hours including vigorous activity

^2^ > 20–80 MET-hours including vigorous activity

^3^ 20–80 MET-hours moderate intensity activity

^4^ < 20 MET-hours

**Table 3:** Physical functioning, emotional wellbeing and workability mean scores (+ 95% CI) by leisure-time physical activity groups with different levels of sitting time

		Model 1	Model 2
Emotional wellbeing			
Low sitting time^1^	Vigorous activity^3^	74.9, 74.2–75.7	74.8, 74.0-75.5
	Moderate activity^4^	72.5, 71.3–73.7	71.8, 70.5–73.0
	Low activity^5^	70.5, 69.1–71.9	71.1, 69.7–72.6
High sitting time^2^	Vigorous activity^3^	72.7, 71.6–73.9	72.9, 71.7–74.1
	Moderate activity^4^	70.4, 68.6–72.1	70.3, 68.6–72.0
	Low activity^5^	67.5, 65.5–69.5	68.0, 66.1–70.0
**Physical functioning**			
Low sitting time^1^	Vigorous activity^3^	96.3, 95.8–96.7	96.0, 95.5–96.4
	Moderate activity^4^	93.9, 93.2–94.6	94.6, 93.8–95.3
	Low activity^5^	92.3, 91.5–93.2	93.3, 92.4–94.1
High sitting time^2^	Vigorous activity^3^	96.1, 95.4–96.8	95.2, 94.5–95.9
	Moderate activity^4^	93.6, 92.6–94.7	93.7, 92.7–94.7
	Low activity^5^	93.2, 91.9–94.2	93.4, 92.3–94.6
**Work ability**			
Low sitting time^1^	Vigorous activity^3^	82.0, 81.3–82.6	81.8, 81.2–82.5
	Moderate activity^4^	79.3, 78.3–80.4	79.3, 77.2–80.4
	Low activity	77.5, 76.3–78.7	78.6, 77.4–79.9
High sitting time^2^	Vigorous activity^3^	80.8, 79.8–81.8	79.9, 78.9–90.0
	Moderate activity^4^	77.6, 76.1–79.1	77.3, 75.8–78.8
	Low activity^5^	77.3, 75.5–79.0	77.4, 75.8–79.1

^1^ ≤ 8.5 h of sitting per weekday

^2^ > 8.5 h of sitting per weekday

^3^ > 20 MET-hour per week including vigorous activity

^4^ > 20 MET-hour per week only moderate activity

^5^ ≤ 20 MET-hour per week physical activity

Model 1: Adjusted for age and sex

Model 2: Model 1 + marital status, having children, education, smoking, binge drinking, BMI, physical and mental strenuousness of work

The joint association analyses showed that higher LTPA was associated with better emotional wellbeing among both high and low sitting time groups, but overall, the high sitting time group had lower emotional wellbeing scores (Table 3). For emotional wellbeing, the low activity group with low (mean score 70.5, 95% CI 69.1–71.9) and high (67.5, 65.5–69.5) sitting times and the moderate activity group with low (72.5, 71.3–73.7) and high (70.4, 68.6–72.1) sitting times had statistically significantly lower mean scores than the corresponding vigorous activity group with low (74.9, 74.2–75.7) and high (72.7, 71.6–73.9) sitting times. LTPA and sitting time showed similar associations to LTPA with physical functioning and work ability scores across different levels of sitting time (Table 3). Physical functioning scores were significantly higher among the vigorous activity group in both low (96.3, 95.8–96.7) and high sitting time groups (96.2, 95.5–96.9) than among the moderate activity groups in the low (93.9, 93.2–94.6) and high (96.1, 95.4–96.8) sitting time groups. The low activity group had the lowest mean scores in both the low (92.3, 91.5–93.2) and high sitting time groups (93.1, 91.9–94.2). For work ability mean scores, the patterns were similar: significantly higher scores were observed for the vigorous activity in both the low (82.0, 81.3–82.6) and high (80.8, 79.8–81.8) sitting time groups. The moderate activity and low activity groups showed significantly lower mean scores in both sitting time groups than the vigorous activity groups. Adjustment for covariates only slightly attenuated these associations.

## Discussion

We examined joint associations of LTPA and sitting time with emotional wellbeing, physical functioning and work ability among 19–39-year-old Finnish municipal employees. The joint analyses showed similar associations of LTPA with functional health outcomes across different levels of sitting time, and those with low LTPA and high sitting times had the poorest emotional wellbeing. However, in physical functioning and work ability, the patterns were similar between the physical activity groups across categories of sitting time. Individual associations showed that higher LTPA was associated with better work ability and physical functioning as well as emotional wellbeing, whereas high sitting time was associated only with poorer emotional wellbeing.

In this cohort of municipal employees aged under 40 years, about 60% were vigorously active (according to our criteria), that is, engaging in more than 20 MET-hours of LTPA per week including vigorous activity equivalent to, for instance, 1 h of jogging and 2 h of brisk walking. Over 20% were moderately active, equivalent to 3.5 h of brisk walking, and just under 20% were classified in the low activity group. Physical functioning scores were high in general, but emotional wellbeing and work ability showed somewhat lower scores.

Among these relatively young and highly functioning, although on average overweight, employees, LTPA showed positive associations with physical functioning and emotional wellbeing. Work ability showed similar associations, which is plausible since physical and mental health functioning are the main contributors to work ability [26]. Vigorous activity showed clear and significant differences with all the examined functional health outcomes, while the moderately active participants had weaker and non-significant differences compared to those who had low activity levels, similar to our previous studies on midlife and older adults [13, 14]. Sitting time showed weak associations with the functional health outcomes, except for some associations with emotional wellbeing, which is consistent with previous studies showing increased depression and anxiety among those with high sedentary time [27, 28]. In this study, we examined total sitting time. Previous studies have shown associations with total sitting time with health, but excess sitting time in different contexts –– such as leisure-time screen time, transport [27] and excess sitting at work [29] –– has also shown independent associations with health. Examining joint associations of leisure-time and commuting physical activity and total sitting time with work ability and physical functioning and emotional wellbeing fills some existing research gaps since previous studies have only examined the individual contributions. Sitting time in different contexts and their associations with functional health outcomes should be examined in more detail in further studies. In particular, when examining occupational cohorts, total sitting time is likely higher among sedentary office workers, however, high sitting at work in the general working population is associated with poorer health, whereas increasing physical workload shows the opposite [30]. Nonetheless, our sensitivity analyses (data not shown) show that those with low sedentary time are more likely to have physical work. Further sensitivity analyses (data not shown), however, showed similar associations with the examined outcomes when excluding occupational sitting from the total sitting time.

The examined joint associations showed poorest functional health outcomes among those with low LTPA and high sitting time, and vice versa, the better health among those with high LTPA and low sitting time. Vigorous activity showed clear differences with all outcomes compared to both inactivity and moderate activity regardless of sitting time, whereas moderate activity showed only modest non-significant differences compared to inactivity. Vigorous activity has been associated with various beneficial health outcomes such as reduced sickness absence [31], lower premature mortality risk [32] and better mental health [14] among older and midlife adults, and these findings extend this to younger employees as well. Vigorous activity likely reflects better physical fitness [33] but may also reflect selection and reverse causality, thus longitudinal studies and randomised controlled trials are needed to confirm these findings.

Employers might consider providing opportunities for active commuting [34] and active mini breaks during workdays [35] since the initiatives could improve the wellbeing and work ability of their employees. The maintenance of work ability over the course of one’s work career is increasingly important for both individuals and the public economy as the old-age dependency ratio has been increasing and is projected to continue increasing in the future [36]. Improving emotional wellbeing and physical functioning are important for the maintenance of work ability and reducing work disability [11]. Based on the present findings as well as previous studies [17], promoting physical activity of the population may prove useful since it is shown to be important for work ability. There is much potential as only a third of the adult Finnish population fulfil the current recommendation for health-enhancing physical activity [37]. Furthermore, cardiorespiratory fitness has been declining for the past 40 years in many countries including Finland [38], probably having negative consequences for work ability [39]. However, we need studies conducted among the general population to confirm the findings and to improve generalisability.

### Methodological considerations

We examined a high functioning large occupational cohort of under 40-year-old municipal employees representing a wide variety of manual and non-manual occupations and including both females and males. Nevertheless, there are some characteristics in our data that limit the generalisability of the findings to the general population. All participants were employed, and 80% of them were females which corresponds to the sex distribution of the municipal sector in general in Finland and the target population. We pooled female and male participants in the analyses, thus these results are female-dominated. The cross-sectional study design is a clear limitation, limiting causal inference since the associations are likely reciprocal, that is, while physical activity and reduced sitting time likely offer health benefits, poorer health and functional limitations also limit participation in physical activities—especially higher intensity activity—and may also result in higher sitting time. Future studies with longitudinal designs need to be conducted. The use of accelerometer measures would have added to the study, however, in large datasets, questionnaires are feasible and widely used, providing reasonably accurate estimates. The physical activity and sitting time questionnaires used have not been validated, however, similar measures are widely used in epidemiological studies and have been shown to be valid for measuring leisure-time and commuting physical activity [40] and sedentary time [41]. Since the health outcomes were also measured with questionnaires, the same source bias may exist. A non-response analysis showed that the data are broadly representative of the target population [20]. Thus, it is unlikely that the non-response substantially biased the examined associations.

## Conclusions

Our findings suggest that high LTPA including vigorous activity is associated with better emotional wellbeing, physical functioning and work ability compared to lower activity levels across different levels of sitting time. Those with low LTPA and high sitting time had the poorest emotional wellbeing. LTPA dominated the associations over sitting time. Physical activity should be promoted among employees since a sufficient amount and intensity of physical activity is likely to contribute to better physical and mental health as well as better work ability.

## Data Availability

The datasets used and/or analysed during the current study are available from the corresponding author on reasonable request.

## Abbreviations

BMI: Body mass index
LTPA: Leisure-time physical activity
MET: Metabolic equivalent
